# Impact of Social Determinants of Health on Health-Related Quality of Life Among Cancer Survivors in the United States

**DOI:** 10.21203/rs.3.rs-4797703/v1

**Published:** 2024-09-02

**Authors:** Josephine Peitz, Michael Zhong, Clement Adebamowo, Sally N. Adebamowo

**Affiliations:** University of Maryland School of Medicine; University of Maryland School of Medicine; University of Maryland School of Medicine; University of Maryland School of Medicine

**Keywords:** Social determinants of health, Health-related quality of life, Cancer, Public health

## Abstract

**Purpose:**

Health-related quality of life (HRQoL) is a critical aspect of cancer survivorship, influenced by various social determinants of health (SDoH) such as economic stability, education access, and healthcare coverage. Understanding the impact of these determinants is essential for developing interventions that improve the well-being of cancer survivors.

**Methods:**

Cross-sectional analyses were conducted using data from 20,534 adults with cancer, including 15,754 from the All of Us (AOU) Research Program (2015–2024) and 4,780 from the National Health and Nutrition Examination Survey (NHANES) (2001–2018). HRQoL outcomes were assessed across multiple dimensions: physical health, mental health, emotional well-being, social support, functional ability, and physical activity.

**Results:**

Higher economic stability, education access, and healthcare coverage were significantly associated with better HRQoL outcomes in both cohorts. In the AOU cohort, those with higher family income were more likely to report very good (OR: 20.24; CI: 12.86–31.87) or excellent (OR: 33.06; CI: 20.01–54.64) quality of life. Similar trends were observed for physical and mental health. The NHANES cohort showed consistent findings. Participants with no negative SDoH factors were significantly more likely to report excellent outcomes across all HRQoL dimensions.

**Conclusions and Implications for Cancer Survivors:**

These findings highlight the significant impact of SDoH on cancer survivors’ HRQoL and support the need for targeted interventions and policies to mitigate the adverse effects of negative SDoH factors. Addressing economic, educational, and healthcare disparities is crucial for improving the long-term health and quality of life of cancer survivors.

## Introduction

Cancer survivorship is increasingly recognized as a distinct and integral phase in the cancer care continuum, which includes prevention, early detection, diagnosis, treatment, and end-of-life care [1]. With advancements in early detection and treatment, the number of cancer survivors has risen substantially. In 2022, is estimated that there are 18.1 million cancer survivors in the United States. This represents approximately 5.4% of the population, and this figure is projected to increase to increase by 24.4%, to 22.5 million, by 2032 [2]. While survival rates have improved, cancer survivors often face long-term physical, psychological, and social challenges that can significantly affect their health-related quality of life (HRQoL). The HRQoL of cancer survivors is influenced by a complex interplay of factors that extend beyond the biological implications of the disease and its clinical management. Issues such as chronic pain, fatigue, mental health disorders, and social isolation are prevalent among this population, making it imperative to address the broader determinants of health that influence these outcomes. Social determinants of health (SDoH) play a crucial role in shaping the health outcomes and overall well-being of individuals.

SDoH are the non-medical factors that influence health outcomes, and include the conditions in which people are born, grow, live, work, and age, as well as the systems put in place to deal with illness [3]. Research has consistently demonstrated that SDoH have a significant impact on the health outcomes of patients with cancer. For instance, socioeconomic disparities have been linked to variations in cancer care, with financial security, lack of insurance, and access to transportation being identified as substantial barriers to optimal health outcomes [4]. Moreover, the availability of cancer clinical trials and the participation of marginalized populations in such trials are also affected by adverse SDoH, such as economic stability and education [5]. Additionally, factors like healthcare access, economic stability, and the neighborhood and built environment have been associated with a lower likelihood of experiencing poor mental and/or physical health among cancer survivors [6, 7]. Understanding the impact of these determinants on cancer survivors is essential for developing targeted interventions and policies aimed at improving their quality of life. Despite the growing recognition of these factors, there is limited research focused on the impact of SDoH on HRQoL among cancer survivors. This study leverages data from two large, nationally representative cohorts to identify specific SDoH factors that have significant impact on HRQoL outcomes among cancer survivors in the United States. The findings from this study would enhance our understanding of the social context of cancer survivorship and inform the development and implementation of effective support strategies.

## Methods

### Study Participants

The study participants were adults enrolled in the All of Us Research Program (AoU) and the National Health and Nutrition Examination Survey (NHANES). The AoU is a longitudinal cohort study aimed at enrolling diverse participants across the United States [8]. We included the baseline data of AOU participants who had completed the study procedures by March 2023, had ever been diagnosed with cancer, and had completed the SDoH surveys.

NHANES uses multistage probability sampling methods to select a series of nationally representative samples of non-institutionalized US adults in 2-year cycles [9]. We included individuals from NHANES who were enrolled in the 2001 to 2018 cycles, had ever been diagnosed with cancer, and had completed the SDoH surveys.

### Ascertainment of Social Determinants of Health

We used the PhenX protocols [10] to identify measures relevant for the collection of comparable SDoH across different datasets and categorized the SDoH measures according to the Healthy People 2030 domains [11]. We included seven available SDoH in AoU and NHANES for economic stability (employment, food security, income), education access and quality (education), and healthcare access and quality (health insurance coverage, health insurance type, and the type of place individuals often go to for healthcare). The ascertainment of the SDoH measures for each cohort is shown in Supplementary Fig. 1.

### Ascertainment of Health-Related Quality of Life

HRQoL were measured as described previously [12]. The ascertainment of the HRQoL measures for each cohort is shown in Supplementary Fig. 2.

In AoU, self-reported quality of life was rated as poor, fair, good, very good, or excellent. Physical health, mental health, social satisfaction, and performance in social roles were reported in the same fashion. Functional ability was categorized by the ability to complete everyday activities: not at all (poor), a little (fair), moderately (good), mostly (very good), and completely (excellent). Emotional well-being was categorized by the frequency of emotional problems in the past week: always (poor), often (fair), sometimes (good), rarely (very good), and never (excellent).

In NHANES, self-reported health SRH was rated as poor, fair, good, very good, or excellent. The physically unhealthy days, mentally unhealthy days, and inactive days variables were categorized into four levels: 0 days (poor), 1–9 days (fair), 10–19 days (good), and 20–30 days (excellent). Social support was assessed by two items: the number of close friends and the frequency of attending church or religious services per year. Scores ranged from 0 (no close friends) to 4 (four or more close friends) for the number of friends, and from 0 (never) to 3 (three or more times) for service attendance. The combined social support score ranged from 0 to 7, categorized as poor (0–1), good (2–4), and excellent (5–7). Emotional well-being was assessed using two questions: availability of emotional support and need for more emotional support in the past year. Scores ranged from 0 (no support or no additional support needed) to 1 (support available or additional support needed). The combined emotional well-being score ranged from 0 (poor) to 2 (excellent).

### Statistical analysis

We used *t*-tests to assess differences in the distribution of continuous variables between groups and χ^2^ and Fisher’s exact tests for categorical variables. We used age-adjusted logistic regression models to investigate the association between each SDoH and HRQoL outcome, using the lowest categories as the reference. Additionally, the models were further adjusted for age at cancer diagnosis, gender and body mass index (BMI). We created a cumulative SDoH score ranging from zero to seven by assigning a value of zero to each favorable and one to each unfavorable SDoH and summing the scores. Because only a small proportion of participants reported more than three unfavorable SDoH, we examined the association between SDoH scores from zero to three or more and several HRQoL outcomes. All the *p* values reported were two-sided. The analyses were performed using SAS 9.4 for UNIX statistical software (SAS Institute, Gary, NC, USA).

### Role of the funding source

The funders did not contribute to the study design, data collection, analysis, interpretation, or report writing.

## Results

A total of 20,534 adults with cancer were included in the analysis, 15,754 from AOU (2015–2024) and 4,780 from the NHANES (2001–2018) cohorts. The characteristics of the study participants at enrolment, are shown in Table 1. The study participants in AOU were older (mean age 70 years vs. 66 years) and their age at cancer diagnosis was higher (60 years, vs 55 years) compared to those in NHANES. The majority of the participants in both study cohorts were female, non-Hispanic whites, not working, had high food security and good healthcare access (Table 2).

### Overall Quality of life and Physical Health

In the AOU cohort, most participants reported their quality of life (42%, 6,654/15,754) and physical health (37%, 5,792/15,754) were very good (Supplementary Table 1). The odds ratios (OR) and 95% confidence intervals (CI) were statistically significant when comparing those with lower economic stability, education access, and healthcare coverage, to those with higher SDoH factors, for both quality of life and physical health. Compared to those with the lowest family income, those with higher family income were more likely to report fair (OR: 2.72; CI: 1.74–4.27), good (OR: 5.81; CI: 3.76–8.98), very good (OR: 20.24; CI: 12.86–31.87), or excellent (OR: 33.06; CI: 20.01–54.64) quality of life, p for trend < 0.001; and fair (OR: 1.92; CI: 1.50–2.45), good (OR: 4.36; CI: 3.41–5.59), very good (OR: 9.98; CI: 7.46–13.36), or excellent (OR: 11.69; CI: 7.64–17.88) physical health, p for trend < 0.001 (Table 3).

In the NHANES cohort, most participants reported their overall health was good (38%, 1,613/4,267) and that they had excellent physical health in the past 30 days (77%, 2,112/2,728). Factors that contribute to economic stability and education access were positively associated with both physical health and health in the past 30 days. All the factors that contribute to healthcare coverage, except for insurance coverage, were significantly associated with overall health and physical health in the past 30 days. Compared to those with no insurance, those with insurance coverage were more likely to report fair physical health (OR: 1.84; CI: 0.61–5.60), good physical health (OR: 0.72; CI: 0.35–1.47), or excellent physical health (OR: 0.93; CI: 0.50–1.74) in the past 30 days, p for trend 0.924 (Table 3).

### Mental, Emotional and Social Wellbeing

In AOU, most participants reported very good mental health (40%, 6,228/15,754), emotional well-being (rarely having emotional problems in the past week) (37%, 5,845/15,754), performance in social roles (38%, 6,032/15,754), and satisfaction with social interactions (39%, 6,184/15,754). In NHANES most participants indicated they experienced excellent mental health (66%, 1,813/2,729), emotional well-being (86%, 717/832), and received substantial social support (58%, 530/921) (Supplementary Table 2). Across both AOU and NHANES cohorts, factors contributing to economic stability and access to education were significantly associated with mental, emotional, and social well-being. However, this association was not significant for some healthcare-related factors (Table 4).

### Functional Ability and Physical Activity

Most participants in AOU reported an excellent ability to perform everyday activities (68%, 10,683/15,754). Employment status, economic stability, food security, and the type of place participants often go to for healthcare were significantly associated with the ability to perform everyday activities (Table 5).

In NHANES, most participants indicated excellent physical activity (no physically inactive days) (77%, 2,112/2,735). All the SDoH factors except the type of place participants often go to for healthcare and insurance coverage status, showed a significant association with physical activity (Table 5).

### SDoH Score

**Figure 1:**
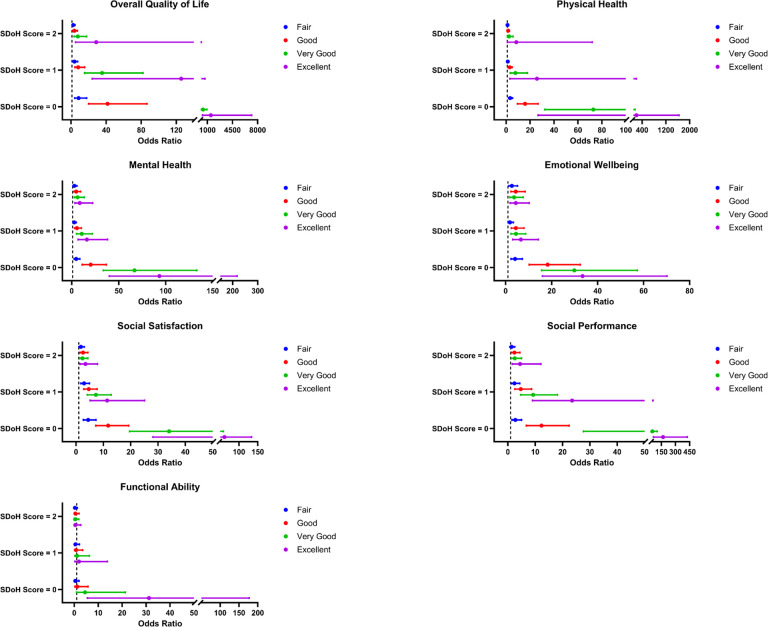
Association of SDoH Score and HRQoL in All of Us Research Program

The distribution of SDoH score in AOU was 82% (12,884/15,754) of participants had none, 13% (2,036/15,754) had one, 4% (661/15,754) had two, and 1% (173/15,754) had at least three negative SDoH factors. In comparison to participants with SDoH score 3+, participants with no negative SDoH were more likely to report excellent quality of life (OR: 1486.70; CI: 307.70–7183.30), excellent physical health (OR: 209.73; CI: 26.74–1644.86), excellent mental health (OR: 93.32; CI: 39.97–217.90), excellent emotional wellbeing (OR: 33.46; CI: 15.95–70.19), excellent social satisfaction (OR: 61.33; CI: 28.13–133.71), excellent performance in social roles (OR: 167.35; CI: 65.83–425.47) and excellent physical activity (OR: 31.21; CI: 5.49–177.28). For stratified analysis, females (p < 0.0001) and other Hispanic (p < 0.0001) were more likely to report 3 + negative SDoH (Fig. 1).

**Figure 2:**
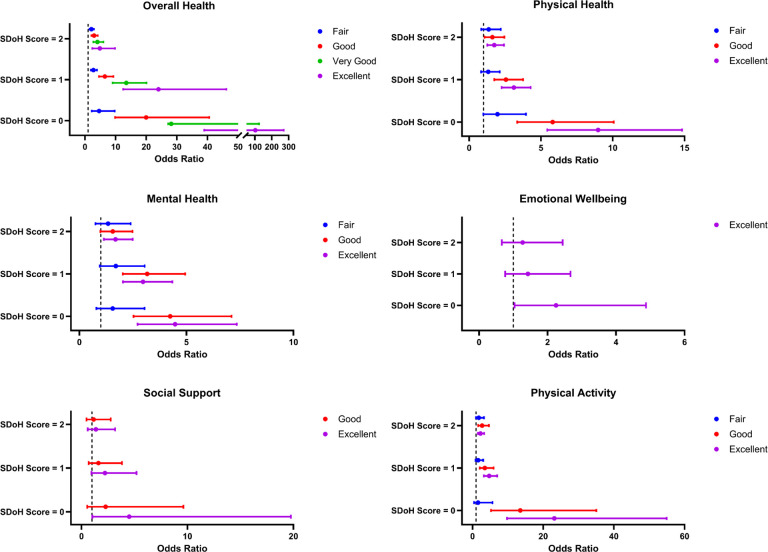
Association of SDoH Score and HRQoL in NHANES

In NHANES, 17% (817/4,780) of participants had none, 40% (1,911/4,780) had one, 26% (1,229/4,780) had two, and 17% (823/4,780) had at least three negative SDoH factors. Compared to those with SDoH score 3+, those with no negative SDoH were more likely to report excellent overall health (OR: 102.74; CI: 38.91–271.25), excellent physical health (OR: 8.98; CI: 5.44–14.82), excellent mental health (OR: 4.47; CI: 2.71–7.35), excellent emotional wellbeing (OR: 2.24; CI: 1.03–4.87), excellent social support (OR: 4.50; CI: 1.02–19.76), and excellent physical activity (OR: 23.14; CI: 9.75–54.95). In stratified analysis, females (p < 0.0001) and other Hispanic, including Mexican American, (p < 0.0001) were more likely to report 3 + negative SDoH (Fig. 2).

SDoH: Social Determinants of Health. HRQoL: Health-Related Quality of Life. The comparison group is SDoH Score = 3+. The models are adjusted for age + age at cancer diagnosis + gender + body mass index.

SDoH: Social Determinants of Health. HRQoL: Health-Related Quality of Life. NHANES: National Health and Nutrition Examination Survey. Not every NHANES survey question had distinct responses for fair/good/very good/excellent, so some responses cannot be depicted. The comparison group is SDoH Score = 3+. The models are adjusted for age + age at cancer diagnosis + gender + body mass index.

## Discussion

This study aimed to examine the impact of SDoH on HRQoL among cancer survivors in the United States, using data from the AOU Research Program and NHANES. The findings revealed significant associations between SDoH factors and various aspects of HRQoL, including physical health, mental health, emotional well-being, social support, and functional ability.

Most of the previous studies have examined HRQoL among cancer survivors but not the impact of SDoH. In the US, previous studies were conducted to examine the HRQoL among cancer survivors, using data from the Behavioral Risk Factor Surveillance System (BRFSS) [6, 13, 14]. Studies conducted with the BRFSS 2000–2002 [14], and 2009 [13] data showed that social and emotional support were associated with overall health, physical health, mental health, and social functioning among adult cancer survivors. Unmet support needs and fear of cancer recurrence were also reported to be significantly associated with physical, functional, and emotional HRQoL domains [15]. A recent study conducted with the BRFSS 2017 and 2019 data showed that cancer survivors who could afford to see a doctor had a decreased risk of poor mental and physical health. Homeowners had a decreased risk of poor mental health [6]. Both unmet support needs and fear of recurrence were significant correlates of physical, functional, and emotional HRQoL domains [15].

In the present study, we observed that economic stability, education access, and healthcare coverage are pivotal in improving the quality of life and physical health of cancer survivors. Participants with higher family incomes reported significantly better outcomes, indicating a positive correlation between financial stability and well-being. This aligns with findings from a which examined socioeconomic differences in HRQoL among cancer survivors and comparison with a cancer-free population, which reported that those with lower socioeconomic status often report impaired quality of life [16]. In the AOU cohort, a notable number of participants described their quality of life and physical health as very good or excellent. In contrast, the NHANES cohort predominantly rated their overall health as good and physical health in the past 30 days as excellent. These variances could be reflective of the different demographic and socioeconomic profiles present in each cohort, such as variations in age and the duration since cancer diagnosis.

The association between social support and quality of life has been well-documented, further emphasizing the need for comprehensive support systems for this population [15, 17]. In our study, we found that participants with better economic stability and access to education reported higher levels of mental health, fewer emotional problems, and greater satisfaction with social interactions. However, the impact of healthcare-related factors on these outcomes was less pronounced, suggesting that while healthcare access is important, other social determinants may play a more critical role in influencing mental and social well-being.

Our findings on functional ability and physical activity levels were similar to those from other studies, which reported that regular physical activity is associated with better physical function and functional capacity among individuals with cancer [18, 19]. We observed that most participants reported excellent ability to perform everyday activities, with significant associations observed across all SDoH factors except the type of usual healthcare facility and type and insurance coverage. This suggests that regular access to comprehensive healthcare services, rather than emergency or urgent care, contributes to better functional outcomes. Furthermore, studies have demonstrated that inadequate health insurance is linked to later-stage cancer diagnoses and poorer survival outcomes, emphasizing the importance of healthcare coverage [20].

Our results showed independent associations of single SDoH factors, and a cumulative effect of multiple unfavorable SDoH on all the HRQoL domains. Participants with no negative SDoH factors were significantly more likely to report excellent outcomes across the HRQoL domains, including quality of life, physical and mental health, emotional well-being, social satisfaction, performance in social roles, and physical activity. The stratified analysis indicated that females and Hispanic individuals, particularly Mexican Americans, were more likely to report multiple negative SDoH factors, suggesting that targeted interventions may be necessary to address disparities in these subgroups.

This study has several limitations. Given the cross-sectional design, reliance on self-reported data and differences in the study cohorts, we were unable to account for changing SDoH measures over time, rule out bias or establish causality between. Additionally, we did not have data on cancer treatment, sufficient power to conduct analyses stratified by the type of cancer and we weighted the seven SDoH equally to generate a cumulative score. Nonetheless, the study utilized a large, diverse sample from two nationally representative cohorts (AOU and NHANES). This extensive dataset enhances the statistical power of the analysis, identifies consistent patterns, and improves the generalizability of the findings across different populations. The comprehensive assessment of multiple SDoH factors and detailed analysis of various HRQoL domains provide a holistic view of the independent and cumulative impact of SDoH on the overall well-being of cancer survivors.

In conclusion, this study highlights the impact of SDoH on HRQoL among cancer survivors in the United States. Economic stability, educational access, and healthcare coverage are crucial for improving physical health and overall well-being. Importantly, financial stability and education were positively associated with greater social satisfaction, better mental health and fewer emotional problems, while healthcare-related factors, although important, appeared to play a less critical role. Future studies could overcome some of the the limitations of this research by utilizing larger sample sizes, longitudinal designs and more objective measures to reduce reliance on self-reported data. Such studies can implement stratified analyses by cancer type, stage at diagnosis, and treatment modalities to uncover specific SDoH impacts unique to different cancer subgroups. Mechanistic studies that explore how SDoH factors impact HRQoL are also warranted. Understanding these pathways can reveal critical intervention points and inform the development of targeted strategies to mitigate the adverse effects of negative SDoH factors.

## Supplementary Material

1

## Figures and Tables

**Table 1:** Characteristics of the Study Participants

Characteristics	AOU	NHANES
	(n = 15754)	(n = 4780)
	Mean (SD)	
**Age**, **years**	70.1 (11.6)	66.2 (14.4)
**Age at diagnosis**, **years**	60.2 (12.1)	55.2 (17.3)
**Body Mass Index**, **kg/m**^**2**^	28.9 (6.4)	28.9 (6.4)
	n (%)	
**Age**		
20–29	50 (0.3)	105 (2.2)
30–39	305 (1.9)	209 (4.4)
40–49	750 (4.8)	353 (7.4)
50–59	1637 (10.4)	618 (12.9)
60–69	4086 (25.9)	1091 (22.8)
70–79	6347 (40.3)	1287 (26.9)
80+	2579 (16.4)	1117 (23.4)
**Gender**		
Male	6307 (40.9)	2247 (47.0)
Female	9051 (58.7)	2533 (53.0)
Other	53 (0.3)	-
**Race/Ethnicity**		
Non-Hispanic White	13246 (87.3)	3314 (69.3)
Non-Hispanic Black	755 (5.0)	675 (14.1)
Other Hispanic	847 (5.6)	567 (11.9)*
Other, including Multiracial	329 (2.2)	224 (4.7)
**BMI, kg/m** ^**2**^		
Underweight (< 18.0)	133 (0.8)	75 (1.7)
Normal Weight (18.1–24.9)	4415 (28.0)	1175 (26.8)
Overweight (25.0–30.0)	5562 (35.3)	1552 (35.4)
Obese (> 30.0)	5644 (35.8)	1584 (36.1)

NHANES: National Health and Nutrition Examination Survey. AOU: All of Us research program. Gender (AOU) – ‘Other’ includes nonbinary, not man only not woman only, transgender, prefer not to answer, and “additional options.”

* Other Hispanic includes Mexican American.

**Table 2:** SDOH Distribution of the Study Participants

SDOH Domains	Characteristics	AOU	NHANES
Economic Stability	**Employment Status**		
	Working	35.5	26.7
	Not Working^a^	64.5	73.3
	**Food Security**		
	Very Low	-	5.4
	Low	1.4	6.8
	Marginal	5.7	7.6
	High	92.8	80.2
	**Family Income** ^b^		
	Low	10.2	29.3
	Medium-Low	15.9	38.8
	Medium	16.8	20.4
	Medium-High	14.8	11.5
	High	42.2	
Education Access and Quality	**Education**		
	< 9th Grade	0.5	10.3
	9th -11th Grade^c^	0.8	12.8
	High School Graduate^d^	8.1	23.0
	Some College/AA	22.5	28.7
	≥ College Graduate	68.1	25.1
Healthcare access	**Healthcare**		
	Hospital ER	0.8	1.7
	Other^e^	4.7	4.6
	Doctor’s Office/HMO	94.5	76.1
	Clinic/Healthcare Center	-	17.6
	**Insurance Coverage**		
	No	1.1	5.9
	Yes	98.9	94.1
	**Insurance Type**		
	Private	50.8	60.5
	Medicare/Medigap	39.2	30.0
	Medicaid/SCHIP	2.5	4.8
	Military	1.1	1.4
	Other^f^	6.4	3.3

NHANES: National Health and Nutrition Examination Survey. AOU: All of Us research program.;

a Not Working (AOU = unable to work, out of work less than one year, out of work one or more years, student, homemaker, retired; NHANES = with job or business but not at work, looking for work, and not working at job or business);

b Family Income (NHANES: low = < 20,000 USD; medium-low = 20,000–44,999 USD; medium = 45,000–74,999 USD; medium-high = 75,000 or more USD);

c 9th -11th Grade (AOU and NHANES = includes 12th grade no diploma);

d High School Graduate (AOU and NHANES = includes GED or equivalent);

e Other, Healthcare (AOU = includes urgent care, some other place, no one place most often; NHANES = includes hospital outpatient department, some other place, and more than one place);

f Other, Insurance Type (AOU and NHANES = includes state-sponsored, other government insurance, and single-service plans).

**Table 3:** Odds Ratios and 95% Confidence Intervals for Physical Health and Overall Quality of life for the Study Cohorts

	All of Us (AOU)					National Health and Nutrition Examination Survey (NHANES)				
	Physical Health					Physical Health				
	Fair (n = 2481)	Good (n = 5294)	Very Good (n = 5792)	Excellent (n = 1652)	p Trend	Fair (n = 119)	Good (n = 303)	Excellent (n = 2112)	P Trend	
Education	1.47 (0.63, 3.39)	2.85 (1.22, 6.70)	15.75 (4.75, 52.20)	13.24 (2.52, 69.67)	0.007	1.12 (0.91, 1.36)	1.38 (1.19, 1.61)	1.33 (1.18, 1.49)	< 0.001	
Employment	2.39 (1.90, 3.02)	7.46 (5.91, 9.40)	18.01 (13.66, 23.75)	19.45 (12.93, 29.26)	< 0.001	1.09 (0.84, 1.42)	1.73 (143, 2.10)	2.00 (1.67, 2.38)	< 0.001	
Family Income	1.92 (1.50, 2.45)	4.36 (3.41, 5.59)	9.98 (7.46, 13.36)	11.69 (7.64, 17.88)	< 0.001	0.90 (0.67, 1.20)	1.45 (1.18, 1.80)	1.53 (1.28, 1.83)	< 0.001	
Food Security	1.34 (0.85, 2.11)	3.73 (2.29, 6.09)	12.06 (6.21, 23.43)	18.67 (5.79, 60.22)	< 0.001	0.97 (0.77, 1.23)	1.43 (1.16, 1.77)	1.72 (1.46, 2.02)	< 0.001	
Healthcare	1.59 (0.98, 2.59)	2.13 (1.31, 3.45)	2.54 (1.50, 4.30)	2.53 (1.28, 5.00)	0.053	1.07 (0.73, 1.57)	0.99 (0.74, 1.31)	1.08 (0.85, 1.37)	0.378	
Insurance Coverage	0.94 (0.44, 2.01)	1.04 (0.49, 2.22)	2.35 (0.97, 5.71)	1.91 (0.55, 6.60)	0.038	1.84 (0.61, 5.60)	0.72 (0.35, 1.47)	0.93 (0.50, 1.74)	0.924	
Insurance Type	1.76 (0.57, 5.43)	3.72 (127, 10.91)	11.13 (2.61, 47.47)	4.70 (0.63, 35.05)	0.014	1.00 (0.81, 1.25)	1.47 (1.20, 1.80)	1.30 (1.15, 1.47)	< 0.001	
	Overall Quality of Life					Overall Health				
	Fair (n = 1073)	Good (n = 3417)	Very Good (n = 6654)	Excellent (n = 6654)	p Trend	Fair (n=948)	Good (n = 1613)	Very Good (n = 1099)	Excellent (n = 306)	P Trend
Education	3.22 (1.03, 10.03)	3.18 (1.10, 9.20)	21.97 (6.20, 77.87)	71.97 (15.68, 330.39)	< 0.001	1.37 (1.22, 1.53)	1.88 (1.68, 2.12)	2.43 (2.14, 2.75)	2.79 (2.33, 3.35)	< 0.001
Employment	2.39 (1.52, 3.77)	6.58 (4.23, 10.22)	21.77 (13.95, 33.97)	33.01 (20.32, 53.64)	< 0.001	1.35 (1.16, 1.56)	1.76 (1.52, 2.03)	2.02 (174, 2.35)	2.28 (1.88, 2.76)	< 0.001
Family Income	2.72 (174, 4.27)	5.81 (3.76, 8.98)	20.24 (12.86, 31.87)	33.06 (20.01, 54.64)	< 0.001	1.35 (1.13, 1.62)	1.85 (1.56, 2.20)	2.45 (2.05, 2.94)	2.68 (2.12, 3.37)	< 0.001
Food Security	1.77 (0.94, 3.30)	5.31 (2.83, 9.95)	29.63 (14.09, 62.33)	36.34 (14.44, 91.44)	< 0.001	1.32 (1.15, 1.50)	1.83 (1.61, 2.08)	2.61 (2.21, 3.08)	4.03 (2.85, 5.70)	< 0.001
Healthcare	1.70 (0.77, 3.74)	2.89 (134, 6.22)	4.03 (184, 8.83)	5.10 (2.26, 11.51)	0.002	1.11 (0.92, 1.33)	1.48 (124, 1.77)	1.82 (1.49, 2.22)	1.88 (142, 2.49)	< 0.001
Insurance Coverage	1.11 (0.33, 3.72)	1.07 (0.33, 3.52)	3.18 (0.93, 10.88)	8.46 (1.89, 37.87)	< 0.001	0.81 (0.48, 1.35)	1.26 (0.76, 2.10)	2.12 (1.19, 3.78)	2.56 (1.13, 5.80)	< 0.001
Insurance Type	4.76 (0.84, 26.92)	3.69 (0.88, 15.50)	24.47 (5.02, 119.18)	39.73 (6.40, 246.79)	0.002	1.15 (1.01, 1.30)	1.31 (117, 1.47)	1.69 (1.46, 1.94)	1.84 (146, 2.31)	< 0.001

The models are adjusted for age + age at cancer diagnosis + gender + body mass index. AOU - Reference groups for HRQOL are: ‘poor’ overall quality of life (n = 103) and ‘poor’ physical health (n = 407). Reference groups for SDOH are: Education: < 9th grade, Employment status: ‘unable to work’ or ‘out of work, Family income: <25,000 USD, Food security: ‘often true’ in response to “Within the past 12 months, were you worried whether your food would run out before you got money to buy more?”, Healthcare: ‘emergency room’ or ‘some other place’ or ‘no one place most often’, Insurance Coverage ‘no insurance’, Insurance type: ‘Medicaid’ or ‘military’. NHANES - Reference groups for HRQOL are: ‘poor’ overall health (n = 301) and ‘poor’ physical health (n = 379). Reference groups for SDOH are: Education: < 9th grade, Employment status: ‘not working with job or business’, Family income: <20,000 USD, Food security: ‘very low’, Healthcare: ‘hospital ER’, Insurance Coverage ‘no insurance’, Insurance type: ‘single service plan’

**Table 4:** Odds Ratios and 95% Confidence Intervals for Mental, Emotional and Social Wellbeing for the Study Cohorts

	All of Us (AOU)					National Health and Nutrition Examination Survey (NHANES)			
	Mental Health					Mental Health			
	Fair (n = 1122)	Good (n = 3328)	Very Good (n = 6628)	Excellent (n = 4825)	p Trend	Fair (n = 158)	Good (n = 495)	Excellent (n = 1813)	p Trend
Education	1.78 (0.59, 5.33)	3.28 (1.12, 9.60)	10.68 (3.23, 35.26)	10.80 (2.92, 39.89)	0.001	1.08 (0.91, 1.27)	1.41 (1.24, 1.59)	1.32 (1.18, 1.47)	< 0.001
EmPloyment	1.76 (1.20, 2.57)	4.22 (2.92, 6.10)	7.91 (5.44, 11.52)	9.25 (6.24, 13.73)	< 0.001	1.06 (0.90, 1.24)	1.32 (1.16, 1.49)	1.31 (1.16, 1.47)	< 0.001
Family Income	1.75 (1.17, 2.62)	3.44 (2.33, 5.06)	7.66 (5.13, 11.44)	7.20 (4.69, 11.06)	< 0.001	1.20 (0.95, 1.50)	1.73 (1.45, 2.07)	1.56 (1.32, 1.83)	< 0.001
Food Security	1.73 (0.96, 3.13)	4.42 (2.44, 8.01)	13.99 (7.08, 27.65)	9.20 (4.37, 19.36)	< 0.001	1.11 (0.91, 1.36)	1.56 (1.31, 1.86)	1.69 (1.45, 1.96)	< 0.001
Healthcare	2.37 (1.26, 4.46)	3.61 (1.98, 6.58)	4.93 (2.67, 9.10)	4.61 (2.43, 8.77)	0.006	1.03 (0.74, 1.43)	1.01 (0.78, 1.30)	0.98 (0.77, 1.24)	0.244
Insurance Coverage	1.62 (0.61, 4.34)	2.10 (0.81, 5.42)	2.40 (0.91, 6.34)	3.71 (1.24, 11.11)	0.018	1.74 (0.86, 3.51)	1.23 (0.74, 2.05)	1.72 (1.07, 2.77)	0.022
Insurance Type	5.00 (0.89, 27.96)	4.71 (1.09, 20.32)	15.42 (3.15, 75.38)	13.38 (2.65, 67.52)	0.017	1.11 (0.91, 1.35)	1.28 (1.10, 1.50)	1.19 (1.05, 1.35)	0.050
	Emotional Wellbeing					Emotional Wellbeing			
	Fair (n = 985)	Good (n = 3964)	Very Good (n = 5845)	Excellent (n = 4678)	p Trend	Excellent (n = 717)			p Trend
Education	0.96 (0.21, 4.36)	1.47 (0.34, 6.46)	2.66 (0.55, 12.93)	2.06 (0.44, 9.52)	0.271	1.16 (0.98, 1.37)			0.081
EmPloyment	2.02 (1.43, 2.85)	4.57 (3.28, 6.37)	8.05 (5.71, 11.34)	8.01 (5.52, 11.64)	< 0.001	1.05 (0.88, 1.26)			0.581
Family Income	1.82 (125, 2.65)	3.69 (2.57, 5.31)	6.04 (4.14, 8.81)	5.24 (3.47, 7.92)	< 0.001	1.24 (0.97, 1.57)			0.082
Food Security	1.60 (0.93, 2.74)	4.24 (2.48, 7.26)	6.47 (3.54, 11.82)	11.92 (5.49, 25.87)	< 0.001	1.71 (1.32, 2.22)			< 0.001
Healthcare	1.90 (102, 3.54)	3.92 (2.14, 7.20)	3.29 (1.79, 6.06)	3.44 (1.79, 6.59)	0.085	1.07 (0.74, 1.54)			0.821
Insurance Coverage	2.14 (0.87, 5.26)	2.38 (1.05, 5.42)	3.80 (1.55, 9.33)	2.37 (0.86, 6.55)	0.033	0.98 (0.42, 2.27)			0.961
Insurance Type	0.66 (0.11, 3.83)	2.08 (0.37, 11.52)	2.26 (0.35, 14.39)	0.72 (0.06, 8.76)	0.064	1.16 (0.92, 1.47)			0.194
	Social Satisfaction					Social Support			
	Fair (n = 1442)	Good (n = 3609)	Very Good (n = 6184)	Excellent (n = 4053)	p Trend	Good (n = 340)	Excellent (n = 530)		p Trend
Education	1.42 (0.45, 4.45)	1.32 (0.46, 3.78)	2.81 (0.92, 8.62)	3.64 (0.99, 13.39)	0.033	1.57 (1.21, 2.04)	1.81 (1.39, 2.35)		< 0.001
Employment	2.02 (1.55, 2.64)	4.00 (3.11, 5.16)	8.11 (6.28, 10.47)	10.44 (7.83, 13.93)	< 0.001	1.01 (0.76, 1.35)	1.20 (0.90, 1.61)		0.020
Family Income	2.18 (1.65, 2.86)	3.48 (2.68, 4.52)	7.80 (5.96, 10.21)	10.20 (7.52, 13.84)	< 0.001	1.67 (1.11, 2.53)	2.00 (1.32, 3.02)		0.003
Food Security	2.64 (1.67, 4.17)	4.39 (2.82, 6.82)	10.95 (6.65, 18.02)	19.31 (9.97, 37.43)	< 0.001	1.58 (1.11, 2.26)	2.01 (1.40, 2.88)		< 0.001
Healthcare	1.40 (0.83, 2.38)	2.10 (126, 3.48)	3.00 (1.82, 4.93)	2.91 (1.70, 4.98)	0.006	0.93 (0.50, 1.73)	1.03 (0.59, 1.79)		0.669
Insurance Coverage	0.81 (0.33, 1.98)	0.93 (0.39, 2.19)	1.30 (0.54, 3.13)	1.95 (0.73, 5.24)	0.016	1.78 (0.59, 5.40)	3.66 (1.19, 11.27)		0.017
Insurance Type	3.71 (1.10, 12.52)	3.46 (1.24, 9.63)	9.07 (3.13, 26.28)	12.97 (3.87, 43.41)	0.009	1.00 (0.65, 1.54)	1.13 (0.79, 1.62)		0.145
	Performance in Social Roles								
	Fair (n = 1027)	Good (n = 2856)	Very Good (n = 6032)	Excellent (n = 5579)	p Trend				
Education	1.39 (0.46, 4.25)	1.94 (0.67, 5.65)	8.80 (2.64, 29.27)	9.43 (2.66, 33.46)	< 0.001				
Employment	1.61 (1.13, 2.29)	4.47 (3.19, 6.25)	12.23 (8.76, 17.07)	21.68 (15.12, 31.09)	< 0.001				
Family Income	1.48 (1.03, 2.12)	2.90 (2.05, 4.10)	7.13 (5.01, 10.14)	11.98 (8.19, 17.50)	< 0.001				
Food Security	1.28 (0.72, 2.28)	2.67 (1.48, 4.80)	7.61 (3.96, 14.62)	16.96 (7.93, 36.23)	< 0.001				
Healthcare	1.96 (1.06, 3.60)	2.96 (1.66, 5.30)	3.84 (2.17, 6.81)	4.45 (2.43, 8.15)	< 0.001				
Insurance Coverage	1.31 (0.49, 3.53)	1.34 (0.52, 3.44)	2.88 (1.10, 7.54)	5.09 (1.82, 14.23)	0.003				
Insurance Type	0.96 (0.17, 5.41)	1.17 (0.24, 5.80)	5.31 (1.01, 27.92)	7.26 (1.13, 46.71)	0.011				

The models are adjusted for age + age at cancer diagnosis + gender + body mass index. AOU - Reference groups for HRQOL are: ‘poor’ self-reported mental health (n = 144), ‘always’ frequency of emotional problems in past 7 days (n = 182), ‘poor’ social satisfaction (n = 339), ‘poor’ self-reported performance in social roles (n = 174). Reference groups for SDOH are: Education: < 9th grade, Employment status: ‘unable to work’ or ‘out of work’, Family income: <25,000 USD, Food security: ‘often true’ in response to “Within the past 12 months, were you worried whether your food would run out before you got money to buy more?”, Healthcare: ‘emergency room’ or ‘some other place’ or ‘no one place most often’, Insurance Coverage ‘no insurance’, Insurance type: ‘Medicaid’ or ‘military’. NHANES - Reference groups for HRQOL are: ‘poor’ mental health (n = 263), ‘poor’ emotional well-being (n = 115), and ‘poor’ social support (n = 51). Reference groups for SDOH are: Education: < 9th grade, Employment status: ‘not working with job or business’, Family income: <20,000 USD, Food security: ‘very low’, Healthcare: ‘hospital ER’, Insurance Coverage ‘no insurance’, Insurance type: ‘single service plan’

**Table 5:** Odds Ratios and 95% Confidence Intervals for Functional Ability and Physical Activity for Each Study Cohort

	All of Us (AOU)					National Health and Nutrition Examination Survey (NHANES)			
	Functional Ability					Physical Activity			
	Fair (n = 704)	Good (n = 1493)	Very Good (n = 2714)	Excellent (n = 10683)	p Trend	Fair (n = 119)	Good (n = 303)	Excellent (n = 2112)	P Trend
Education	-	-	-	-	-	1.12 (0.91, 1.36)	1.38 (1.19, 1.61)	1.33 (1.18, 1.49)	< 0.001
Employment	1.31 (0.70, 2.46)	2.36 (1.31, 4.26)	6.10 (3.46, 10.77)	18.32 (10.63, 31.58)	< 0.001	1.09 (0.84, 1.42)	1.73 (1.43, 2.10)	2.00 (1.67, 2.38)	< 0.001
Family Income	1.31 (0.70, 2.45)	1.74 (0.95, 3.17)	3.89 (2.15, 7.04)	9.47 (5.20, 17.24)	< 0.001	0.90 (0.67, 1.20)	1.45 (1.18, 1.80)	1.53 (1.28, 1.83)	< 0.001
Food Security	0.41 (0.10, 1.76)	0.74 (0.17, 3.14)	1.07 (0.24, 4.80)	3.20 (0.58, 17.60)	< 0.001	0.97 (0.77, 1.23)	1.43 (1.16, 1.77)	1.72 (1.46, 2.02)	< 0.001
Healthcare	0.89 (0.26, 3.03)	1.23 (0.37, 4.06)	2.10 (0.63, 6.96)	2.88 (0.89, 9.38)	< 0.001	1.07 (0.73, 1.57)	0.99 (0.74, 1.31)	1.08 (0.85, 1.37)	0.378
Insurance Coverage	-	-	-	-	-	1.84 (0.61, 5.60)	0.72 (0.35, 1.47)	0.93 (0.50, 1.74)	0.924
Insurance Type	-	-	-	-	-	1.00 (0.81, 1.25)	1.47 (1.20, 1.80)	1.30 (1.15, 1.47)	< 0.001

The models are adjusted for age + age at cancer diagnosis + gender + body mass index. AOU - Reference groups for HRQOL are: ‘not at all’ ability to perform everyday activities (n = 61); Reference groups for SDOH are: Education: < 9th grade, Employment status: ‘unable to work’ or ‘out of work’, Family income: <25,000 USD, Food security: ‘often true’ in response to “Within the past 12 months, were you worried whether your food would run out before you got money to buy more?”, Healthcare: ‘emergency room’ or ‘some other place’ or ‘no one place most often’, Insurance Coverage ‘no insurance’, Insurance type: ‘Medicaid’ or ‘military’. NHANES - Reference groups for HRQOL are: ‘poor’ physical activity (n = 201); Reference groups for SDOH are: Education: < 9th grade, Employment status: ‘not working with job or business’, Family income: <20,000 USD, Food security: ‘very low’, Healthcare: ‘hospital ER’, Insurance Coverage ‘no insurance’, Insurance type: ‘single service plan’

## Data Availability

NHANES data are available on their website https://wwwn.cdc.gov/nchs/nhanes/Default.aspx and All of Us data are available on their researcher workbench after undergoing registration and training: https://www.researchallofus.org/data-tools/workbench/.
