# Supplementary material for: Impact of Social Determinants of Health on Health-Related Quality of Life Among Cancer Survivors in the United States
Source: Res Sq. 2024 Sep 2:rs.3.rs-4797703. Preprint. [Version 1] doi: 10.21203/rs.3.rs-4797703/v1 (PMC11398579; doi:10.21203/rs.3.rs-4797703/v1)
Supplement: 1 [file NIHPPrs4797703v1-supplement-1.pdf]

Supplementary Fig. 1. Key SDOH measures in AOU and NHANES			
Domain	Primary Measure	Ascertainment	
Economic Stability	Employment	AOU	What is your current employment status? Please select 1 or more of these categories.
		NHANES	Which of the following were you doing last week: working at a job or business, with a job or business but not at work, looking for work, or not working at a job or business?
	Food insecurity	AOU	1. Within the past 12 months, we worried whether our food would run out before we got money to buy more.<br><br>2. Within the past 12 months, the food we bought just didn't last and we didn't have money to get more.<br><br>Possible answers: Often true, sometimes true, never true, skip
		NHANES	Eighteen Food Security Survey Module questions were asked of households with children under the age of 18. Ten questions were asked of households without children. A categorical variable for household food insecurity was created based on the number of affirmative responses to those questions.
	Income	AOU	What is your annual household income from all sources?
		NHANES	The respondent was asked to report total family income for themselves and the other members of their family in dollars.
Education Access and Quality	Education	AOU	What is the highest grade or year of school you completed?
		NHANES	What is the highest grade or level of school you have completed or the highest degree you have received?
Healthcare access	Health insurance coverage	AOU	Are you covered by health insurance or some other kind of health care plan?
		NHANES	Are you covered by health insurance or some other kind of health care plan? [Include health insurance obtained through employment or purchased directly as well as government programs like Medicare and Medicaid that provide medical care or help pay medical bills.]
	Health insurance type	AOU	Are you currently covered by any of the following types of health insurance or health coverage plans? Select all that apply from one group.
		NHANES	Are you covered by: private insurance, Medicare, Medi-Gap, Medicaid, SCHIP, military health care, state-sponsored health plan, other government insurance, or single service health plan?
	Type of place most often go for healthcare	AOU	Is there a place that you USUALLY go to when you are sick or need advice about your health? What kind of place do you go to most often?
		NHANES	What kind of place do you go to most often when you are sick or need advice about your health. Is it: a clinic or health center, doctor's office or HMO, hospital emergency room, hospital outpatient department, or some other place?

Ascertainment of Health-Related Quality of Life

HRQoL were measured as described previously [12]. The ascertainment of the HRQoL measures for each cohort is shown in Supplementary Fig. 2.

Supplementary Fig. 2. Key HRQOL measures in AOU and NHANES		
Primary Measure	Ascertainment	
Self-Reported Quality of Life (AOU), Self-Reported Health (NHANES)	AOU	In general, would you say your quality of life is:
	NHANES	I have some general questions about {your/SP's} health. Would you say {your/SP's} health in general is. . .
Physical Health	AOU	In general, how would you rate your physical health?
	NHANES	Thinking about your physical health, which includes physical illness and injury, for how many days during the past 30 days was your physical health not good?
Mental Health	AOU	In general, how would you rate your mental health, including your mood and your ability to think?
	NHANES	Now thinking about your mental health, which includes stress, depression, and problems with emotions, for how many days during the past 30 days was your mental health not good?
Emotional Wellbeing	AOU	In the past 7 days, how often have you been bothered by emotional problems such as feeling anxious, depressed or irritable?
	NHANES	Emotional well-being was also examined using two questions: whether there was anybody to help with emotional support, and if more emotional support was needed in the last year. If the participant answered they did not have anyone to help with emotional support a score of 0 was assigned, and a score of 1 was given if they answered yes. If the participant answered they did not need more emotional support in the last year, a score of 0 was given, and if they needed more support a score of 1 was assigned. Emotional well-being then had a scale of 0 for ‘low’ emotional well-being, 1 for ‘mid’ emotional well-being, and 2 for ‘high’ emotional well-being.
Social Satisfaction (AOU), Social Support (NHANES)	AOU	In general, how would you rate your satisfaction with your social activities and relationships?
	NHANES	Social support was assessed by the two items: number of close friends and how often they attend a church or religious service per year. A score was created then where no close friends was assigned a score of 0, 1 close friend was 1, 2 close friends was 2, 3 close friends was 3, and 4 or more close friends was 4. Yearly church/other religious service attendance was score so ‘never’ was scored 0, ‘once’ was scored 1, ‘twice’ was score 2, and 3 or more times was score 3. Participants could then have a social support score from 0 to 7, which we grouped into 3 categories: 0–1 was considered ‘low’ social support, 2–4 was ‘mid’ social support, and 5–7 was ‘high’ social support.
Performance in Social Roles	AOU	In general, please rate how well you carry out your usual social roles. (This includes activities at home, at work and in your community, and responsibilities as a parent, child, spouse, employee, friend, etc.)
	NHANES	N/A
Functional Ability and Physical Activity	AOU	To what extent are you able to carry out your everyday physical activities such as walking, climbing stairs, carrying groceries, or moving a chair?
	NHANES	During the past 30 days, for about how many days did poor physical or mental health keep you from doing your usual activities, such as self-care, work, school or recreation?

In AoU, self-reported quality of life was rated as poor, fair, good, very good, or excellent. Physical health, mental health, social satisfaction, and performance in social roles were reported in the same fashion. Functional ability was categorized by the ability to complete everyday activities: not at all (poor), a little (fair), moderately (good), mostly (very good), and completely (excellent). Emotional well-being was categorized by the frequency of emotional problems in the past week: always (poor), often (fair), sometimes (good), rarely (very good), and never (excellent).
